# Inducible Caspase9-mediated suicide gene for MSC-based cancer gene therapy

**DOI:** 10.1038/s41417-018-0034-1

**Published:** 2018-06-29

**Authors:** Filippo Rossignoli, Giulia Grisendi, Carlotta Spano, Giulia Golinelli, Alessandra Recchia, Giulia Rovesti, Giulia Orsi, Elena Veronesi, Edwin M. Horwitz, Massimo Dominici

**Affiliations:** 1 Division of Oncology, Department of Medical and Surgical Sciences for Children & Adults, University-Hospital of Modena and Reggio Emilia, Modena, Italy; 2 Rigenerand srl, Modena, Italy; 3 Department of Life Sciences, University of Modena and Reggio Emilia, Modena, Italy; 4 Technopole of Mirandola TPM, Modena, Italy; 5 Aflac Cancer and Blood Disorders Center, Department of Pediatrics, Children’s Healthcare of Atlanta, Emory University, Atlanta, GA USA

**Keywords:** Genetic engineering, Cell delivery, Targeted therapies

## Abstract

Cellular therapies based on mesenchymal stromal/stem cells (MSC) are promising strategies in regenerative medicine and oncology. Despite encouraging results, there is still some level of concerns on inoculating MSC in cancer patients. To face this issue, one possibility resides in engineering MSC by incorporating a suicide gene in order to control their fate once infused. Strategies based on Herpes Simplex Virus Thymidine Kinase (HSV-TK) and the Cytosine Deaminase genes have been developed and more recently a novel suicide gene, namely, iCasp9, has been proposed. This approach is based on a variant of human Caspase9 that binds with high affinity to a synthetic, bioinert small molecule (AP20187) leading to cell death. Based on this technology so far marginally applied to MSC, we tested the suitability of iCasp9 suicide strategy in MSC to further increase their safety. MSC have been transfected by a lentiviral vector carrying iCasp9 gene and then tested for viability after AP20187 treatment in comparison with mock-transfected cells. Moreover, accounting our anti-tumor approaches based on MSC expressing potent anti-cancer ligand TNF-Related Apoptosis-Inducing Ligand (TRAIL), we generated adipose MSC co-expressing iCasp9 and TRAIL successfully targeting an aggressive sarcoma type. These data show that anti-cancer and suicide mechanisms can coexist without affecting cells performance and hampering the tumoricidal activity mediated by TRAIL. In conclusion, this study originally indicates the suitability of combining a MSC-based anti-cancer gene approach with iCasp9 demonstrating efficiency and specificity.

## Introduction

Mesenchymal stromal/stem cells (MSC) are a heterogeneous population of fibroblast-like cells originally isolated from bone marrow and other tissues including adipose tissue, peripheral blood, umbilical cord blood, and Wharton’s jelly, among others [1, 2]. MSC retain therapeutic potential in several pathological conditions through different mechanisms such as differentiation into mature cells, secretion of cytokines, and release of microvesicles [3, 4]. Moreover, growing evidences revealed that MSC retain unique immunological features that are relevant for the treatments of immune-related disorders [5] and for their possible use as cellular vehicles to deliver bioactive molecules inside tumor parenchyma and stroma [6]. In particular, this latter possibility gained a relevant interest in the last decade and we and others have been developing anti-tumor approaches based on MSC expressing the potent anti-cancer ligand TNF-Related Apoptosis-Inducing Ligand (TRAIL), variants demonstrating efficacy against several tumors [7–9, Spano et al., Submitted].

Despite encouraging results, there is some level of concern regarding the safety of inoculating both wild-type and gene-modified MSC in particular concerning their possible damage to normal organs, malignant transformation, and promotion of cancer growth [10]. One possibility to face these possibilities is to incorporate a suicide gene in MSC; the most commonly used are the Herpes Simplex Virus Thymidine Kinase and the Cytosine Deaminase [11]. These transgenes confer the ability to convert a non-toxic prodrug into an active cytotoxic compound that kills the cell itself and the neighbors. This “bystander effect” has been exploited to specifically deliver cytotoxic compounds into tumor burdens with therapeutic benefit limiting possible off target toxicity [12]. However, these systems demonstrated a number of disadvantages starting from their possible immunogenicity with consequent elimination by immune system [13] and the high cell-cycle dependency of the activated drugs that limits killing capacity to dividing cells [14]. Additional drawbacks may also come from the limitation in concurrent administration of drugs (i.e., Ganciclovir) that, delivered for a possible CMV-viral infection, can lead to unintended elimination of gene-modified cells [15]; further evidences indicate also the possible onset of drug resistance on target cells [16].

In recent years, alternative strategies have been developed to overcome these limitations. In particular, investigators described a novel suicide gene, namely, iCasp9, based on the sequence of human Caspase9 encoding for a modified protein having a high affinity to a synthetic, bioinert small molecule (B/B Homidimerizer, AP20187) resulting in iCasp9 dimerization and activation eventually leading to cell death [15]. This approach offers several advantages over the other suicide strategies, such as the human origin of the protein, as favorable condition to avoid immunogenicity. Moreover, the suicide trigger is independent from the cell cycle phase and does not interfere with any drug schedule [15]. Several studies showed the high efficacy and specificity of iCasp9 [10, 17, 18] and a safety study on healthy volunteers demonstrated the feasibility of the treatment with the dimerizer molecule in humans [19]. Based on this background, we challenge this approach on anti-cancer MSC delivering TRAIL to generate a proof of concept that would ultimately increase the safety of our cell therapy approach.

## Materials and methods

### Cell culture

The human embryonic kidney cell line 293T and the human Ewing sarcoma cell line A673, both from our laboratory, were maintained in culture at 37 °C in Dulbecco’s Modified Eagle’s Medium (Gibco, Thermo Fisher Scientific Inc., Waltham, MA, USA) supplemented with 10% fetal bovine serum (PAA Laboratories Inc., Etobicoke, Canada), 1% penicillin/streptomycin (10,000 U/mL Penicillin, 10 mg/mL Streptomycin in 0.9% NaCl solution, PAA Laboratories Inc.). Adipose-derived MSC (AD-MSC) were obtained as previously described from lipoaspirate specimens of individuals undergoing liposuction for esthetic purposes after approval by local Ethical Committee [7]. Cells from two different donors were used as biological replicates. After isolation, cells were grown in α-MEM (Gibco) containing 2.5% human platelet lysate (Modena Policlinic Blood Bank, Modena, Italy), 1% L-Glutamine (200 mM in 0.85% NaCl solution, BioWhittaker, Lonza, Verviers, Belgium), 0.5% Ciprofloxacin (Fresenius Kabi Italia S.r.l., Verona, Italy), 1 IU/ml Heparin (Sigma-Aldrich, St. Louis, MO, USA). When confluent, the adherent AD-MSC cells were detached with trypsin/EDTA (Trypsin 0.05% EDTA 0.02% in PBS, EuroClone, Milan, Italy), counted and seeded at 6000 cells/cm^2^. Cells were incubated and maintained within a controlled atmosphere (5% CO_2_ and temperature of 37 °C).

### Vector generation and MSC transduction

The pMSCV-F-del Casp9.IRES.GFP plasmid was obtained from Addgene repository (Addgene plasmid #15567, http://www.addgene.org/). iCasp9 gene was subcloned into a third-generation lentiviral backbone (pCCL.PGK.WPRE) and the resulting construct was transiently transfected into 293T cells together with a mixture of helper plasmids according to jetPEI^®^ protocol (Polyplus Transfection, Illkirch, France). After 48 h, conditioned medium containing lentiviral particles was collected and used to transduce AD-MSC, thus obtaining AD-MSC iCasp9. AD-MSC expressing TRAIL (AD-MSC TRAIL) from our laboratory [7] have been further engineered in the same way to express iCasp9 gene (AD-MSC TRAIL-iCasp9). AD-MSC transduced with the empty lentiviral backbone were used as control (AD-MSC EMPTY).

### Dose-response apoptosis induction assay

Modified AD-MSC were tested for apoptosis induction after the addition of B/B Homodimerizer (AP20187, Clontech Laboratories, Inc., Mountain View, CA, USA) by MTS metabolic assay (CellTiter^®^ 96 AQueous One Solution Cell Proliferation Assay, Promega Corporation, Madison, WI, USA). Briefly, for each tested sample and dimerizer concentration, 5000 cells were seeded in a well of a 96-wells plate. The following day, medium was replaced with fresh one containing different concentrations of B/B Homodimerizer (0.01 nM, 0.1 nM, 1 nM, 10 nM, 100 nM), and after 24 h the plate was analyzed with a Multiskan FC Microplate Photometer (Thermo Fisher Scientific Inc.), according to manufacturer’s instructions.

### AD-MSC TRAIL-iCasp9 cytotoxicity assay

AD-MSC TRAIL-iCasp9 have been then tested for cytotoxic activity against the A673 cell line through a ^51^Cr release assay in coculture and compared with AD-MSC TRAIL and AD-MSC EMPTY. Briefly, tumor cells were labeled with ^51^Cr and then cultured alone or together with AD-MSC, AD-MSC TRAIL, or AD-MSC TRAIL-iCasp9 for 8 and 24 h at several ratio of target:effector cells (1:1, 1:2, 1:5). ^51^Cr released has been detected by 2450 microplate counter MicroBeta2^TM^ (PerkinElmer, Waltham, MA, USA). The experiment layout comprises a maximum ^51^Cr release condition obtained by lysing ^51^Cr-marked cells with Triton X-100 (Sigma-Aldrich) and a background assessment obtained by measuring the beta emission of untreated ^51^Cr-marked cells. The background level is subtracted from all the measures, while the maximum release becomes the reference for 100% mortality in respect of which all the other death rates are calculated. In addition, to test whether unaffected cells developed resistance against the B/B Homodimerizer treatment, we proceeded with a three-hit suicide induction assay. AD-MSC EMPTY and iCasp9 were seeded in three 96-well plates corresponding to the conditions tested and incubated with 10 nM B/B Homodimerizer (1st hit). After 24 h mortality rate was assessed in one of the plates by MTS metabolic assay, while the others were incubated again with 10 nM dimerizer (2nd hit). After additional 24 h, the second plate was evaluated while the remaining one was induced for the last time (3rd hit) for 24 h.

### AD-MSC TRAIL-iCasp9 combined assay

Cytotoxicity and suicide assays were combined together in a comprehensive assay to test whether AD-MSC TRAIL-iCasp9 could be induced to suicide after cytotoxic effect. A673 cells were seeded in 12-well multiwell plate at a density of 6000 cells/cm^2^. The following day, AD-MSC EMPTY, AD-MSC TRAIL, and AD-MSC TRAIL-iCasp9 were stained with CFSE (CellTrace™ CFSE Cell Proliferation Kit, Thermo Fisher Scientific Inc.), according to manufacturer’s protocol and seeded in coculture with A673 cells at 12,000 cells/cm^2^ (1:2 A673:MSC ratio). After 24 h of coculture, 10 nM B/B Homodimerizer was added and induction lasted 18 h. At that point cells were stained with propidium iodide 50 µg/mL (stock solution 1 mg/ml in PBS, Sigma-Aldrich) for 30 min, detached and then the percentage of dead cells was detected by flow cytometry (FACS Aria III, BD Biosciences, San Diego, CA, USA). In particular, apoptotic MSC were identified as both CFSE and PI positive, while A673 apoptotic cells were CFSE negative and PI positive.

### Statistical analyses

All experiments have been performed three times with a technical triplicate. Data have been analyzed using Microsoft Excel 2010 (Microsoft Corporation, Redmond, WA, USA) and are expressed as mean values ± SD unless otherwise noted. Normal distribution of data have been tested using Shapiro–Wilk normality test. Unpaired two-tailed Student’s *t*-test was used considering *p* ≤ 0.05 as statistically significant. ANOVA test was performed using Prism software (GraphPad Software Inc., La Jolla, CA, USA) considering *p* ≤ 0.05 as statistically significant.

## Results

### AD-MSC expressing iCasp9 efficiently undergo apoptosis after induction

**Fig. 1:**
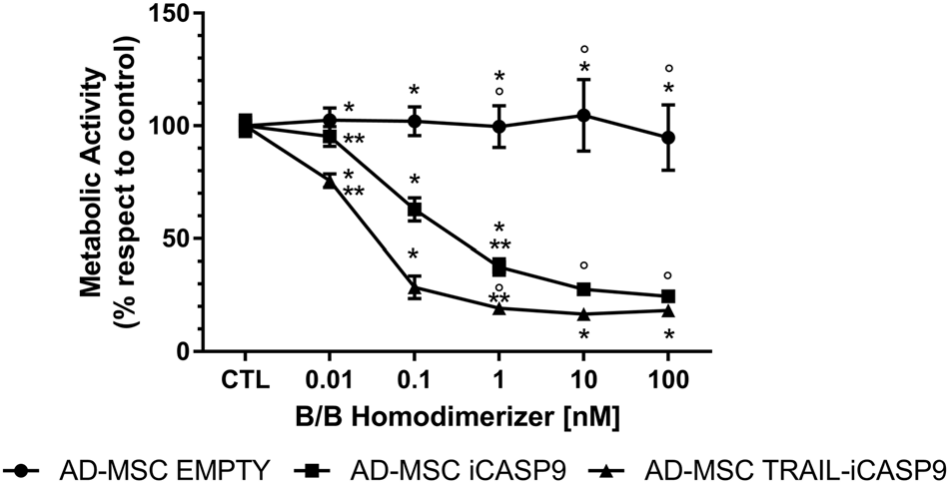
AD-MSC expressing iCasp9 efficiently undergo apoptosis after treatment with B/B Homodimerizer. Dose-response assay in MTS showed the effect on vitality of 24 h incubation with several B/B Homodimerizer concentrations. We observed a progressive drop in cell viability of both AD-MSC iCasp9 and TRAIL-iCasp9 reaching 20% at 100 nM. While the slope is more pronounced for AD-MSC TRAIL-iCasp9, both cell populations reached a similar plateau at 10 nM. The treatment had no effect on AD-MSC Empty. °*p* < 0.0001, *p < 0.0001, **p < 0.01 by ANOVA and multiple comparison tests

Dose-response assay showed that the addition of B/B Homodimerizer efficiently triggers apoptosis in both AD-MSC iCasp9 and AD-MSC TRAIL-iCasp9 (Fig. 1). In particular, after 24 h of treatment, we observed an initial drop in cell viability with concentration of dimerizer as low as 0.01 nM. The increase in concentration corresponded to a decrease in cell viability, reaching 18.3 ± 0.5% for AD-MSC TRAIL-iCasp9 at the 100 nM concentration. Noticeably, apoptosis for both AD-MSC iCasp9 and AD-MSC TRAIL-iCasp9 cells reached a plateau at 1 nM with no difference due to the presence of the TRAIL gene. In the same experimental setting, EMPTY-modified cells were not affected by the presence of dimerizer molecule in all the tested concentrations and time points.

### AD-MSC TRAIL-iCasp9 retain cytotoxic potential against Ewing Sarcoma cell line

**Fig. 2:**
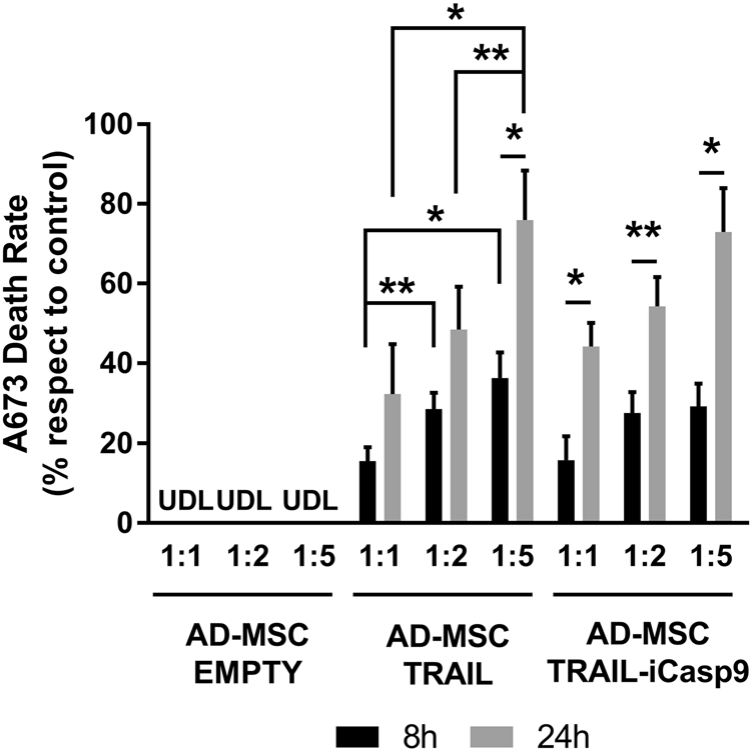
iCasp9 expression does not impair TRAIL-mediated anti-sarcoma cytotoxicity. The ^51^Cr release coculture assay showed a similar cytotoxic capacity of AD-MSC TRAIL and TRAIL-iCasp9. In particular at 1:1 ratio, death rate in coculture with AD-MSC TRAIL increased from 15.5 ± 3.5% after 8 h to 32.4 ± 12.3% after 24 h, at 1:2 ratio increased from 28.6 ± 4.1% after 8 h to 48.5 ± 10.7% after 24 h, and at 1:5 ratio increased from 36.3 ± 6.5% after 8 h to 76.0 ± 12.4% after 24 h. Similarly, in coculture with AD-MSC TRAIL-iCasp9, at 1:1 ratio death rate increased from 15.8 ± 6.1% after 8 h to 44.2 ± 5.9% after 24 h, at 1:2 ratio increased from 27.7 ± 5.2% after 8 h to 54.3 ± 7.3% after 24 h, and at 1:5 ratio increased from 29.3 ± 5.6% after 8 h to 72.9 ± 11.0% after 24 h. UDL UnDetectable Lysis. *p < 0.01, **p < 0.05 by Student’s *t*-test. Data are expressed as mean ± SEM

The ^51^Cr release coculture assay demonstrated that iCasp9 expression does not affect AD-MSC TRAIL cytotoxic activity. As shown in Fig. 2, AD-MSC expressing both iCasp9 and TRAIL are able to kill A673 Ewing Sarcoma cells with an efficacy very similar to that of AD-MSC expressing TRAIL only. The effect has been clearly appreciable even starting from 8 h of culture and becoming more prominent after 24 h. Moreover, we observed an upward trend in MSC cytotoxic capacity as the ratio between tumor cells and MSC decreases, reaching 72.9 ± 11.0% at the lowest T:E ratio of 1:5.

### Surviving AD-MSC iCasp9 maintain sensitivity for the dimerizing treatment

**Fig. 3:**
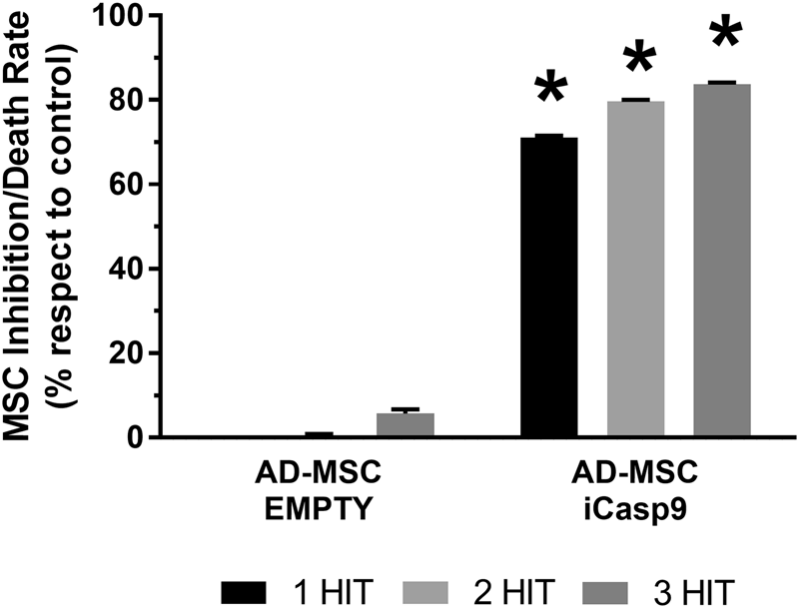
Treatment can progressively eliminate AD-MSC iCasp9. AD-MSC EMPTY or iCasp9 have been treated with 10 nM B/B Homodimerizer from 1 to 3 times. The first 24 h of incubation led to 71.1 ± 0.4% of cell apoptosis and a subsequent identical treatment on survived cells led to 79.7 ± 0.3% cell death. Futhermore another 24 h treatment improved suicide rate up to 83.8 ± 0.3%. These data show that treatment can be repeated to eradicate still alive cells. *p < 0.01 by Student’s *t*-test. Data are expressed as mean ± SEM

AD-MSC iCasp9 surviving after 24 h incubation with a first round of B/B Homodimerizer were again treated by the same protocol to assess whether AD-MSC iCasp9 gained resistance. Interestingly, repeated hits showed an augmented mortality rate at each treatment which increases from 71.1 ± 0.4 to 83.8 ± 0.3%, excluding the development of a resistance (Fig. 3).

### AD-MSC TRAIL- iCasp9 can be induced to apoptosis after death induction in sarcoma cells

**Fig. 4:**
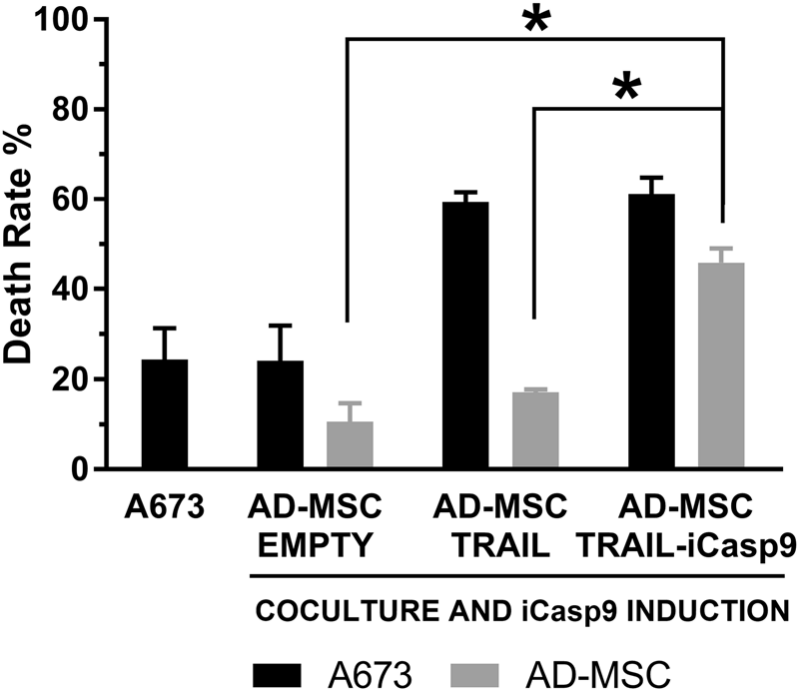
AD-MSC TRAIL-iCasp9 can be prompted to apoptosis after cytotoxic action. Modified MSC were first engaged against tumor cells and then induced to suicide after the completion of cytotoxic activity. This assay confirmed the cytotoxic action of TRAIL-expressing MSC with 59.4 ± 2.2% and 61.2 ± 3.6% A673 cell death in coculture with AD-MSC TRAIL and TRAIL-iCasp9, respectively (A673 basal death rate: 24.1 ± 6.9%) and, from the other side, it recapitulates the proposed approach in which iCasp9-expressing MSC can be committed to apoptosis (45.9 ± 3.1%). *p < 0.01 by Student’s *t*-test

To in vitro validate our suicide strategy, gene-modified AD-MSC were cultured with A673 and then induced to suicide after their cytotoxicity effect had taken place. As visible in Fig. 4, both A673 alone and AD-MSC EMPTY, as controls, showed expected mortality levels with no impact of the dimerizer in both cell types. A673 cultured with AD-MSC TRAIL showed high levels of apoptosis with no effect of the dimerizer on AD-MSC TRAIL. Very interestingly, the AD-MSC TRAIL-iCasp9 confirmed their sarcoma-killing capacity followed by their induction to apoptosis by the dimerizing agent. Collectively these findings indicated that our strategy to induce cancer cell apoptosis by MSC and then eliminate the effector cells is feasible.

## Discussion

Theoretically, cell-based therapies require transplanted cells to survive until the stabilization of their therapeutic function. However, the ability of controlling in the long-term unwanted behaviors of successfully transplanted cells still represent a critical point in safety management. Although MSC have been administered to hundreds of patients without notable side effects, some level of concerns persists especially in regard to gene-modified cells [10]. Thus, to improve the safety of our cell therapy approach we introduced, within a gene-modified MSC platform, a novel suicide mechanism based on an engineered form of Caspase9 which is activated only in presence of a specific dimerizer molecule. This safety mechanism hold promises for an almost complete elimination of modified cells in case of adverse reactions after infusion. This technology was first described by Straathof and colleagues followed by other groups which demonstrated its potential for the elimination of modified T lymphocytes in preclinical [14, 15, 20] and clinical [18] scenarios. However, to the best of our knowledge, studies on its application on MSC is only limited to unmodified bone marrow-derived MSC [10]. Here, we are originally presenting a suicide gene strategy in combination with a TRAIL gene delivery approach for cancer. By this strategy we were able to induce cancer cell death up to 80% in 24 h. While this impact is relevant, we are currently exploring optimized strategies to increase the mortality possibly by the combination of synergizing agents to MSC-delivered TRAIL, as we previously showed using bortezomib against a poorly TRAIL-sensitive triple-negative breast cancer [7] and as more broadly reported by Trivedi et al. [21].

Testing the approach on AD-MSC gene modified to express iCasp9 construct we observed a remarkable rate of cell elimination after the addition of the dimerizer molecule with almost 80% of iCasp9 expressing MSC eliminated after a single dose of dimerizer. On contrary, unmodified cells are not affected by the treatment, thus confirming its specificity. We further include the iCasp9 system in our gene-modified MSC expressing TRAIL and verified that the cytotoxic activity is not hampered by the presence of iCasp9 gene. In particular, we observed a similar cytotoxic activity between MSC TRAIL and TRAIL-iCasp9, in line with our previous observations in vitro [7], thus demonstrating the feasibility of the combined approach. Moreover, the double infection was not interfering with iCasp9-mediated clearance of modified cells and in fact we still observed approximately 85% of cell death in double-infected cells. Nonetheless, an optimization of transfection technology with an improved selection of gene-modified MSC will rather be desirable to more solidly prompt this technology into clinical studies, as already performed for T lymphocytes-based therapies [18]. In the MSC and cancer arena, Neiss et al. pre-clinically gene modified human MSC by a vector containing the HSV-TK gene [22]. In that case transduction efficacy was between 2–30% and required a puromycin treatment. After the selection they were able to safely treat six patients with advanced, recurrent, or metastatic gastrointestinal or carcinoma within a phase I/II study [23]. An increase up to 100% in the level of apoptosis in target cells after activation of the suicide mechanism would also be desirable, although a substantial therapeutic benefit in patients affected by graft-versus-host disease has been achieved even when 90% of T lymphocytes have been killed [18].

Potential limitations of this strategy include the occurrence of spontaneous dimerization of iCasp9 causing undesirable cell death. Although this cannot be excluded in advance, our and other’s observation of low spontaneous death rate argues against the relevance of this point [10]. Another potential limit is the development of resistance due to the upregulation of anti-apoptotic effectors. Nonetheless the involvement of Caspase9 occurs late in the apoptotic pathway and it should therefore bypass the effects of these regulators. We glanced at this issue by verifying that cells surviving to the first administration of dimerizer do not develop a resistance to the treatment and can be killed by a subsequent administration, thus ensuring the reliability of the approach. With the limitation of an in vitro study, these data represent proof-of-concept that it is possible to modulate the survival of gene-modified MSC influencing their tumor killing ability increasing the efficacy of the approach with the release of anticancer agent within the tumor microenvironment and at the same time to control the possible side effects within more efficient and safe cancer gene therapy strategies.
